# Prediction of Ventricular Tachycardia One Hour before Occurrence Using Artificial Neural Networks

**DOI:** 10.1038/srep32390

**Published:** 2016-08-26

**Authors:** Hyojeong Lee, Soo-Yong Shin, Myeongsook Seo, Gi-Byoung Nam, Segyeong Joo

**Affiliations:** 1 Department of Biomedical Engineering, University of Ulsan College of Medicine, Seoul, Republic of Korea; 2 Department of Biomedical Informatics, Asan Medical Center, Seoul, Republic of Korea; 3 Department of Internal Medicine, Asan Medical Center, University of Ulsan College of Medicine, Seoul, Republic of Korea; 4 Department of Biomedical Engineering, Asan Medical Center, Seoul, Republic of Korea

## Abstract

Ventricular tachycardia (VT) is a potentially fatal tachyarrhythmia, which causes a rapid heartbeat as a result of improper electrical activity of the heart. This is a potentially life-threatening arrhythmia because it can cause low blood pressure and may lead to ventricular fibrillation, asystole, and sudden cardiac death. To prevent VT, we developed an early prediction model that can predict this event one hour before its onset using an artificial neural network (ANN) generated using 14 parameters obtained from heart rate variability (HRV) and respiratory rate variability (RRV) analysis. De-identified raw data from the monitors of patients admitted to the cardiovascular intensive care unit at Asan Medical Center between September 2013 and April 2015 were collected. The dataset consisted of 52 recordings obtained one hour prior to VT events and 52 control recordings. Two-thirds of the extracted parameters were used to train the ANN, and the remaining third was used to evaluate performance of the learned ANN. The developed VT prediction model proved its performance by achieving a sensitivity of 0.88, specificity of 0.82, and AUC of 0.93.

Sudden cardiac death (SCD) causes more than 300,000 deaths annually in the United States^1^. Coronary artery disease, cardiomyopathy, structural heart problems, Brugada syndrome, and long QT syndrome are well known causes of SCD^1^,^2^,^3^,^4^. In addition, spontaneous ventricular tachyarrhythmia (VTA) is a main cause of SCD, contributing to about 80% of SCDs^5^. Ventricular tachycardia (VT) and ventricular fibrillation (VF) comprise VTA. VT is defined as a very rapid heartbeat (more than 100 times per minute), which does not allow enough time for the ventricles to fill with blood between beats. VT may terminate spontaneously after a few seconds; however, in some cases, VT can progress to more dangerous or fatal arrhythmia, VF. Accordingly, early prediction of VT will help in reducing mortality from SCD by allowing for preventive care of VTA.

Several studies have reported attempts at predicting VTAs by assessing the occurrence of syncope, left ventricular systolic dysfunction, QRS (Q, R, and S wave in electrocardiogram) duration, QT (Q and T wave) dispersion, Holter monitoring, signal averaged electrocardiograms (ECGs), heart rate variability (HRV), T wave alternans, electrophysiologic testing, B-type natriuretic peptides, and other parameters or method^6^,^7^,^8^,^9^,^10^. Among these studies, prediction of VTAs based on HRV analysis has recently emerged and shown potential for predicting VTA^11^,^12^,^13^.

Previous studies have focused on the prediction of VT using HRV analysis. In addition, most studies assessed the statistical value of each parameter calculated on or prior to the VT event and parameters of control data, which were collected from Holter recordings and implantable cardioverter defibrillators (ICDs)^12^,^14^,^15^. However, the results were not satisfactory in predicting fatal events like VT.

To make a better prediction model of VT, it is essential to utilize multiple parameters from various methods of HRV analysis and to generate a classifier that can deal with complex patterns composed of such parameters^7^. Artificial neural network (ANN) is a valuable tool for classification of a database with multiple parameters. ANN is a kind of machine learning algorithms, which can be trained using data with multiple parameters^16^. After training, the ANN calculates an output value according to the input parameters, and this output value can be used in pattern recognition or classification. ANN has not been widely used in medical analysis since the algorithm is not intuitive for physicians. However, utilization of ANN in medical research has recently emerged^17^,^18^,^19^. Our group previously reported an ANN-based prediction model for VTAs by utilizing parameters obtained from HRV analysis^7^. The model uses RR intervals within a 5-minute window and predicts VT events 10 seconds ahead of the events. The developed prediction model showed 73.3% sensitivity, 73.8% specificity and 75.6% accuracy, which is favorable compared to the performances of previously reported predictors^13^. However, predicting VT 10 seconds before it occurs is not sufficiently valuable in clinical practice. The forecast time was limited to 10 seconds in our previous study because of the length of the RR interval database. The database contains only 1024 RR intervals before VT events, which is about 6 to 12 minutes in time length. Short-term HRV analysis requires at least a 5-minute window, which greatly reduced the forecast time of VT events. To predict VT events earlier than in our previous work, gathering physiological signals including ECG from patients for a longer time period prior to VT events is essential.

In our current study, we propose a model to predict VT events 1 hour in advance by utilizing ANN and parameters from HRV and RRV analysis. Such a model may help to reduce mortality from VT events.

## Results

Before training ANNs, HRV and RRV parameters of VT and control groups were compared to determine statistical differences for each parameter. Mean and standard deviation (SD) were calculated as well as corresponding p-values (Table 1). Standard deviation of NN intervals (SDNN), standard deviation 2 (SD2), and respiration period variability (RPdV) were found to be statistically significant (p <0.05). In contrast, these did not show statistical difference in previous studies^7^.

**Figure 1:**
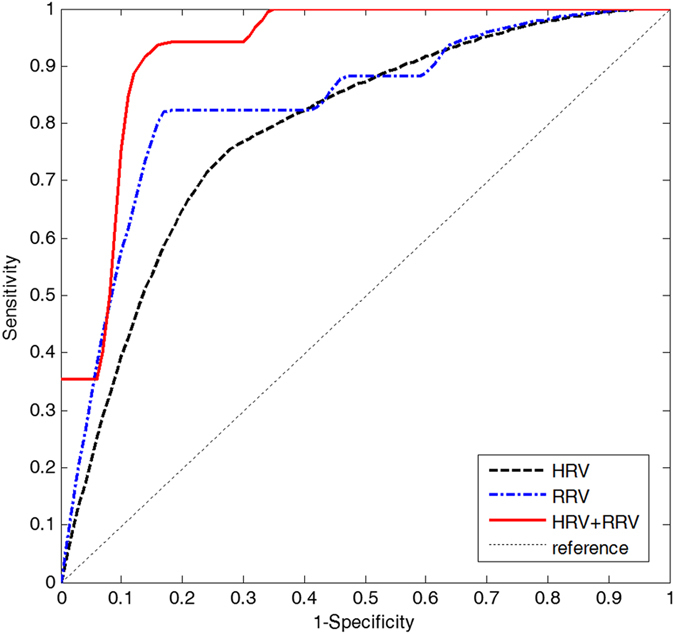
ROC curve of three ANNs (dashed line, with only HRV parameters; dashdot line, with only RRV parameters; solid line, with HRV and RRV parameters; dotted line, reference) used in the prediction of a VT event one hour before onset.

Initially, ANN with only 11 HRV parameters was developed. In our simulation, an ANN with 5 hidden neurons in one hidden layer showed the best performance. After training, the ANN showed 73.5% accuracy for the test set. The sensitivity, specificity, positive predictive value (PPV), and negative predictive value (NPV) were 70.6% (12/17), 76.5% (13/17), 75.0% (12/16), and 72.2% (13/18), respectively. The results are summarized in Table 2. The receiver operating characteristic (ROC) curve of the ANN is shown in Fig. 1, with an area under curve (AUC) of 0.75.

We then developed an ANN with 3 RRV parameters. An ANN with 5 hidden neurons in one hidden layer showed the best performance. The trained ANN showed 82.4% accuracy for the test set. The sensitivity, specificity, PPV, and NPV were equal to 82.4% (Table 2). The AUC of the ROC curve was 0.83 (Fig. 1). The overall performance of the ANN using RRV parameters was found to be superior to that of the ANN using HRV parameters.

Finally, an ANN was generated using all parameters of the HRV and RRV analyses. This ANN with 13 hidden neurons in one hidden layer showed the best performance. The trained ANN showed 85.3% accuracy for the test set. The sensitivity, specificity, PPV, and NPV were 88.2% (15/17), 82.4% (14/17), 83.3% (15/18), and 87.5% (14/16), respectively (Table 2). The AUC of the ROC curve was 0.93 (Fig. 1). This model achieved the best performance among the three ANNs.

## Discussion

As indicated in Table 1, the standard deviation of NN intervals (SDNN), standard deviation 2 (SD2), and respiration period variability (RPdV) were statistically significant (p < 0.05). Other parameters did not show a strong statistical significance between the VT and control dataset. However, one cannot compare the VT and control group based only on these variables. The ranges in SDNN, SD2, and PRdV values determined by the mean and SD values range of the two groups overlaps a little. Therefore, one cannot simply distinguish the two groups with only SDNN, SD2, and RPdV values. In such conditions, machine-learning based classifier is a suitable solution for developing predictors.

The results demonstrate that utilizing both ECG and respiration signals increases the performance of detecting VT one hour before its occurrence. Previously reported studies on the prediction of VT and other arrhythmias used statistical index of ECGs^20^,^21^ or only HRV and heart rate parameters^12^,^13^,^14^,^15^. Cappiello *et al.* showed good performance (accuracy of 98.44%) in prediction of VTA before about 356 ECG beats (about 3 to 5 minutes) but they used only 32 ECGs to develop and verifying their prediction method^21^. One study that focused on using HRV parameters included parametric changes in HRV, such as rise in heart rate, descent in LF, rise in LF/HF, rise in VLF, and reduction in HF observed prior to the VT event^12^. Other studies utilized point estimates of the correlation dimension^14^, slope reduction in heart rate turbulence^15^, and heart rate acceleration^13^. However, these models failed to show any significant performance. The classifier developed by Thong and Paitt showed 91% specificity, but the sensitivity was only 53 ~ 69% using 208 records from 90 subjects^13^. While the classifier developed by Skinner showed good performance (91% sensitivity and 85% specificity), only 14 VT subjects were included in their study^14^.

In contrast, the proposed ANN with HRV and RRV parameters achieved an AUC of 0.93 (88.2% sensitivity and 82.4% sensitivity) using 52 VT cases. The prediction performance of the developed predictor with only HRV showed an AUC of 0.75, which is similar to the VT prediction we previously reported. This implies that prediction of VT with only HRV parameters does not yield sufficient predictive performance. Physicians report that signs of panting or breathing difficulty frequently precede VT occurrence^22^. Furthermore, correlations between sleep apnea and cardiac arrhythmias have been described^23^. One paper reported that sleep-apnea increases the risk of atrial fibrillation^24^. Therefore, including RRV parameters may be important in predicting a VT event.

In addition, the model we have implemented shows great value in predicting VT one hour before its occurrence. The majority of the aforementioned studies reported predictions at or a few minutes before onset. As such, our model would allow for more time to respond to arrhythmia events. If VT leads to VF and ventricular circulation is not properly supplied to the body, organs can undergo damage, which decreases patient survival rate. Therefore, prompt medical action is necessary. One hour may be enough time to visit the hospital if there is no one around to help; as such, this model may be suitable for the general population at home as well as those in hospital.

The major limitation of this study is the small number of data (total: 104 recordings), which limits the statistical power of our analysis. Although we collected the data for about 2 years using 15 patient monitors, we saw a small numbers of actual VT cases. We are currently trying to expand the real-time vital signal collection system to other ICUs to acquire more VT cases. In addition, we will try to combine more signals, such as blood pressure or photo plethysmography. We believe that this could help in developing better predictor of VTs.

Nowadays, wearable devices, such as smart watches, smart necklaces, and smart bands that measure heart rate and other parameters are emerging. Measuring respiratory rate is a challenging issue for such wearable smart devices, especially smart watches, but this may be overcome in the near future. One major goal of such devices is personal healthcare. The developed algorithm can be easily loaded on such devices to predict and warn of fatal arrhythmias in advance.

## Methods

### Real-time acquisition of vital signals

**Figure 2:**
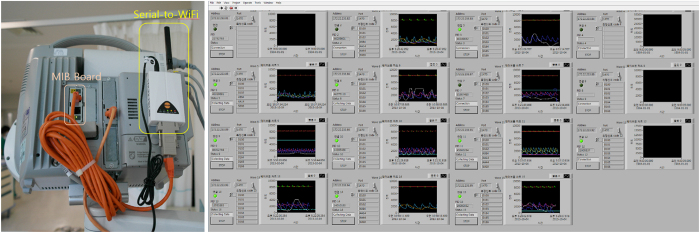
Real-time data acquisition system constructed for real-time acquisition of vital signs. (Left) Photograph of the rear of a patient monitor with a data export board and serial-to-Wi-Fi adaptor. (Right) Data collection software for remote monitoring and storing the collected vital signs.

Real-time physiological monitoring data used in this study was collected from 15 patient monitors (IntelliVue MP40, Phillips, Netherlands) at the cardiovascular intensive care unit (CCU) at Asan Medical Center (AMC). Every measuring signal from patient monitors was digitized and wirelessly transferred to a data server. To achieve this wireless transmission of real-time signals, a data export board (IntelliVue medical information bus/RS232 interface, Phillips, Netherlands) and a serial-to-Wi-Fi interface system (CSW-H80, Sollae System, Korea) were installed on every patient monitor in the CCU. With this configuration, the server can communicate wirelessly with each patient monitor and request alarms and vital signals that the monitor then measures, such as ECG and respiratory rate. Software for the communication with patient monitors, storing data, and data review was implemented using LabVIEW (National Instrument). The software was developed with a programming guide by Philips^25^. The constructed real-time collection system of vital signs is shown in Fig. 2. The institutional review board at the AMC approved the acquisition of data and all other experiments with the waiver of consent (No. 2013–0634). All experiments were performed in accordance with the approved guideline and regulations.

### Database

**Figure 3:**
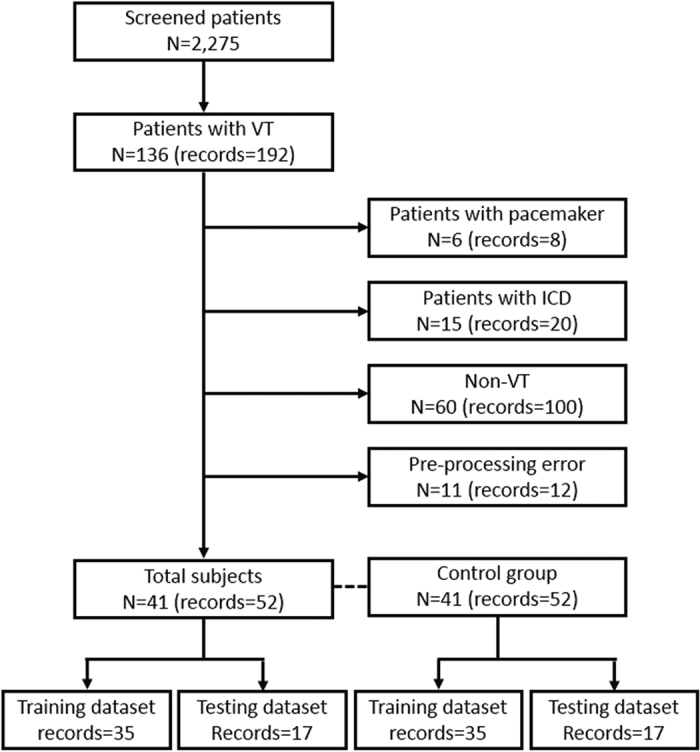
Data selection process used to build the VT database.

The data acquisition server stored every vital signal from 15 patients in the CCU at AMC from September 2013 to April 2015. The total number of patients who visited the CCU within this period was 2,275. To build a VT database using the above raw data, the software obtains the event time and the monitor number when any patient monitor detects a VT. Each time the VT alarm was detected in the database, lead II ECG and respiration signals of 5-minute duration at the point of the VT event (from 5 minutes before the VT event to the VT event) and those of 1 hour before the VT event (from 65 minutes before the VT event to 6o minutes before VT event) were extracted. Data from patients with a pacemaker or ICD were excluded from the VT database because heart rhythm can be controlled by these devices. To remove false positives, ECG signals at onset of the VTs were independently reviewed by two experienced physicians (MSS, GBN). Signals collected at 1 hour before VTs were checked after removing false alarms. If there was a missing record or when a fatal error on the signals was detected, the case was also removed from the VT database. To build a control dataset, 5-minute-long ECG and respiration signals were collected when the patient was stable before and after 12 hours from the collecting point. Finally, the VT database consisted of 52 recordings prior to one hour from VT occurrence and 52 control recordings (total: 104 recordings). The data collection process is shown in Fig. 3.

### Preprocessing and parameter extraction

Short-term HRV analysis was conducted on ECG signals from the database. Likewise, RRV analysis was also done by using respiratory signals from the database. To perform HRV analysis, R-peaks were detected first from the collected ECG using the detection algorithm developed by Sergey Chernenko^26^. Respiratory signals also underwent peak detection with the peak-detection algorithm by Eli Billauer^27^. Before extracting the HRV parameter, ectopic beats were removed as in a previous study^7^. The integrated pulse frequency modulation model^28^ was applied to handle the ectopic beats in the database. After revising ectopic beats, the time domain parameter and Poincaré nonlinear parameter were extracted. The frequency domain parameters were extracted by detrending the RR interval using time-varying finite impulse response high-pass filter after resampling at 7 Hz and cubic spline interpolation^29^. Power spectral density (PSD) of the resampled data was computed using Welch’s periodogram with a 512 points Hann window overlapped at 50%^30^. The spectral power was then calculated at a very low frequency (VLF, 0–0.04 Hz), low frequency (LF, 0.04–0.15 Hz), and high frequency (HF, 0.15–0.4 Hz). In extracting RRV parameters, the respiration signal was filtered with a band-pass filter of 0.1–0.5 Hz to remove noise. After that, the respiratory rate was calculated by measuring the distance between the time domain at positive peaks^31^. Three parameters of respiration period mean (RPdM), respiration period standard deviation (RPdSD), and respiration period variability (RPdV) were then calculated as described in a previous study^32^. Detailed description of other parameters used the study are summarized in Table 3.

### Artificial neural network training

After extracting the 14 parameters from the database as in Table 3, two-thirds of the data (total 70 - VT: 35, control: 35) randomly selected from the complete dataset were utilized to train ANN for classification of VT events (training set). The remaining one-third of the data (total 34 - VT: 17, control: 17) was used to evaluate the performance of the trained ANN (test set).

A back propagation learning rule and a perceptron structure were used in training of ANN. The output value in response to input was set to 1 in the VT group and −1 in the control group. Training was stopped when the mean square error fell below 10^−5^. Finally, three ANNs (trained with parameters of HRV, RRV, and HRV + RRV, respectively) were determined, and the performance of each model was validated. Signal processing and ANN procedures were implemented directly with MATLAB 2012 (MathWorks).

## Additional Information

**How to cite this article**: Lee, H. *et al.* Prediction of Ventricular Tachycardia One Hour before Occurrence Using Artificial Neural Networks. *Sci. Rep.* **6**, 32390; doi: 10.1038/srep32390 (2016).

## Figures and Tables

**Table 1:** Comparison of HRV and RRV parameters between the control and VT dataset.

Parameters	Control dataset (n = 110)	VTs dataset (n = 110)	
	Mean ± SD	Mean ± SD	p-Value
Mean NN (ms)	0.709 ± 0.149	0.718 ± 0.158	0.304
SDNN (ms)	0.061 ± 0.042	0.073 ± 0.045	0.013
RMSSD (ms)	0.068 ± 0.053	0.081 ± 0.057	0.031
pNN50 (%)	0.209 ± 0.224	0.239 ± 0.205	0.067
VLF (ms^2^)	4.1E-05 ± 6.54E-05	6.23E-05 ± 9.81E-05	0.057
LF (ms^2^)	7.61E-04 ± 1.16E-03	1.04E-03 ± 1.15E-03	0.084
HF (ms^2^)	1.53E-03 ± 2.02E-03	1.96E-03 ± 2.16E-03	0.088
LF/HF	0.498 ± 0.372	0.533 ± 0.435	0.315
SD1 (ms)	0.039 ± 0.029	0.047 ± 0.032	0.031
SD2 (ms)	0.081 ± 0.057	0.098 ± 0.06	0.012
SD1/SD2	0.466 ± 0.169	0.469 ± 0.164	0.426
RPdM (ms)	2.73 ± 0.817	2.95 ± 0.871	0.038
RPdSD (ms)	0.721 ± 0.578	0.915 ± 0.868	0.075
RPdV	28.4 ± 5.31	25.4 ± 3.56	<0.002

**Table 2:** Performance of three ANNs in predicting a VT event 1 hour before onset for the test dataset.

ANN with	Input	Sensitivity (%)	Specificity (%)	Accuracy (%)	PPV (%)	NPV (%)	AUC
HRV parameters	11	70.6(12/17)	76.5(13/17)	73.5(25/34)	75.0(12/16)	72.2(13/18)	0.75
RRV parameters	3	82.4(14/17)	82.4(14/17)	82.4(28/34)	82.4(14/17)	82.4(14/17)	0.83
HRV + RRV parameters	14	88.2(15/17)	82.4(14/17)	85.3(29/34)	83.3(15/18)	87.5(14/16)	0.93

**Table 3:** Detailed description of the 11 HRV and 3 RRV parameters used in this study.

Signal	Method	Parameter	Unit	Description
HRV	Time domain analysis	Mean NN	ms	Mean of NN interval
		SDNN	ms	Standard deviation of NN intervals
		RMSSD	ms	Square root of the mean squared differences of successive NN intervals
		pNN50	%	Proportion of interval differences of successive NN intervals greater than 50 ms
	Frequency domain analysis	VLF	ms^2^	Power in very low frequency range (0–0.04 Hz)
		LF	ms^2^	Power in low frequency range (0.04–0.15 Hz)
		HF	ms^2^	Power in high frequency range (0.15–0.4 Hz)
		LF/HF		Ratio of LF over HF
	Poincaré nonlinear analysis	SD1	ms	Standard deviation of points perpendicular to the axis of lineofidentity, or Standard deviation of the successive intervals scaled by 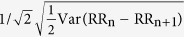
		SD2	ms	Standard deviation of points along the axis of lineofidentity, or 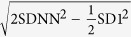
		SD1/SD2		Ratio of SD1 over SD2
RRV	Time domain analysis	RPdM	ms	Respiration period mean (Mean of positive peaks interval in respiration signal, or mean inspiration time)
		RPdSD	ms	Respiration period standard deviation (Standard deviation of positive peaks interval in respiration signal, or the fluctuation rate of the inspiration time)
		RPdV		Respiration period variability (converting ratio of RPdSD over RPdM into a percentage) 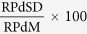
